# Individual variation in breeding phenology and postnatal development in northern bats (*Eptesicus nilssonii*)

**DOI:** 10.1002/ece3.70324

**Published:** 2024-10-23

**Authors:** Mari Aas Fjelldal, Jeroen van der Kooij

**Affiliations:** ^1^ Finnish Museum of Natural History University of Helsinki Helsinki Finland; ^2^ Nature Education, Research and Consultancy van der Kooij Slattum Norway

**Keywords:** Chiroptera, gestation, neonatal, parturition, reproduction

## Abstract

Bats inhabiting northern latitudes are faced with short reproductive seasons during which they must produce and rear pups before fattening up in time to survive the winter hibernation. Therefore, the timing of parturition has considerable impacts on future fitness prospects for the mother and pup. However, little is known about individual variation in breeding phenology and its consequences for postnatal development within bat populations. Here, we studied the phenology of breeding in *Eptesicus nilssonii* across 7 years using data collected by day‐to‐day monitoring of a breeding colony in Norway (60.1° *N*) for which the identity and age of each mother (*N* = 8) and pup (*N* = 28) were known. Using mixed‐effect models, we found that arrival at the colony was influenced by temperature conditions from mid‐April to mid‐May across all females, but that there were strong and consistent individual differences in arrival‐ and parturition time across years. Females generally arrived ~32.1 days before giving birth, but the gestation duration was reduced if females arrived late and prolonged if females left the colony when faced with cold weather conditions. Pups born later in the season were born smaller but had higher growth rates during the most rapid growth period (<10 days old). The within‐individual effects suggest that the higher growth rates could be due to mothers compensating (e.g. through increased food intake) for late parturition rather than by improved food availability. Date of parturition did not influence adult body size in pups. Pups became volant at the earliest only 13.1 days after birth and approached adult flight patterns during their first flight week. Our results suggest that *E. nilssonii* is highly adapted to a short breeding season by producing large, fast developing and early volant pups, despite the environmental pressures bats face at northern latitudes.

## Graphical abstract

Here, we studied individual‐level breeding phenology and postnatal development in *Eptesicus nilssonii* across 7 years, using data collected by day‐to‐day monitoring of a breeding colony in Norway. We show evidence of strong and consistent maternal differences in breeding phenology in bats across years. Our results further suggest that *E. nilssonii* is highly adapted to a short breeding season by producing large, fast‐developing, and early‐volant pups despite the environmental pressures bats face at northern latitudes.

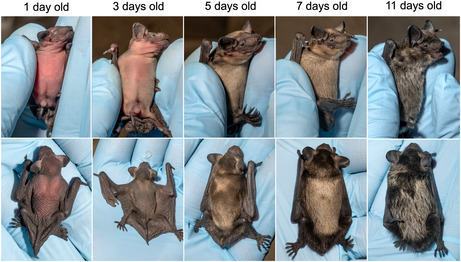

## 1 INTRODUCTION

For many species in seasonal environments, spring and summer are a critical time of year when resource abundance and warmer thermal environments allow individuals to invest in reproduction (Fretwell, 1972). However, at northern latitudes, the spring season can be inhospitable, while the summer season is short. The timing and success of reproduction in high‐latitude environments, therefore, tend to fluctuate annually and largely depend on spring and summer conditions (e.g., Fjelldal et al., 2020; Linton & Macdonald, 2018).

Female bats at northern latitudes follow a reproductive cycle where they mate in autumn, store sperm in the uterus throughout the winter hibernation, and initiate fertilization and gestation in spring before reaching parturition in late spring or early summer (Racey, 1982). Because shorter summer seasons limit the time available for fostering pups and rebuilding fat reserves for the upcoming winter hibernation, reproductive females and their offspring generally benefit from earlier parturition dates in seasonal environments (Frick et al., 2010; Linton & Macdonald, 2018; Ransome & McOwat, 1994). However, females that initiate gestation early may obtain earlier parturition dates, but risk giving birth and entering the energetically costly lactation period before food availability has stabilized (Rydell, 1989a). Pregnant bats, therefore, need to balance the risk of facing an energetic mismatch by giving birth early against the costs associated with giving birth late, both of which could impact individual long‐term fitness.

The trade‐offs associated with the timing of parturition is naturally strongly dependent on interannual spring conditions, where cold, wet, and windy weather conditions can delay the breeding phenology in bats (e.g., Linton & Macdonald, 2018; Sunga et al., 2023). During poor environmental conditions in spring, pregnant females can reduce energetic costs by employing torpor, although this pauses the fetal development and prolongs gestation (Racey & Swift, 1981; Willis et al., 2006). The influence of spring conditions on the timing of parturition in bats is therefore likely caused by a combination of delayed emergence from hibernation sites and increased use of daily torpor during the gestation period. However, after accounting for environmental effects, we still lack knowledge on differences in individual reproductive strategies, or “personalities” (Dingemanse et al., 2010), in insectivorous bats, which potentially exist along a behavioral continuum with two extremes: initiate gestation early to prolong the breeding season and cope with a more stochastic environment, or initiate gestation late during more stable conditions and cope with a limited breeding season and reduced potential to build up fat reserves for the winter. Interannual individual differences in parturition time were detected in *Myotis lucifugus*, where some individuals were consistently later or earlier across years compared to other females in the colony (Sunga et al., 2023). However, these observations were few compared to the overall number of individuals in the study.

For bats belonging to the family of Vespertilionidae, which is the largest family within the order of Chiroptera, the pups are born large (neonatal forearm length ranging from 25% to 43% of maternal forearm length; Kurta and Kunz (1987)) and express rapid early development rates compared to what would be expected from the low annual recruitment and high longevity (Barclay et al., 2003). In northern latitude environments with short breeding seasons, mammals are expected to adopt fast postnatal growth rates to enhance survival chances across the approaching winter season (Boyce, 1979). Observations of postnatal development in bats across latitudes align with these predictions, where a strong selection pressure for faster postnatal growth rates was found in pups born in temperate areas compared to tropical climates (Kunz & Stern, 1995). Breeding colonies of northern bats (*Eptesicus nilssonii*) are found as far north as 69° N (Frafjord, 2013; Rydell et al., 1994; Suominen et al., 2022), which expose them to prolonged periods of continuous daylight under the midnight sun. Being the northernmost breeding bat species in the world, *E. nilssonii* can be expected to possess behavioral, physiological and/or life‐history adaptations that allow them to survive at latitudes that are too challenging for most other bat species (e.g., Fjelldal et al., 2023; Sørås et al., 2023). However, the exact mechanisms allowing this species to overcome the energetic restrictions of costly breeding in such areas are still unknown.

Here, we explore individual‐based data across seven consecutive years (2017–2023) of a small maternity colony of *E. nilssonii* in Norway. Such interannual individual‐based data are scarce within the order of Chiroptera (but see Linton & Macdonald, 2018; Sunga et al., 2023), and detailed day‐to‐day monitoring of mothers and pups have not previously been recorded in free‐ranging bats. In this study, we investigate individual‐level breeding phenology and postnatal development in a colony faced with the challenges of living in a high‐latitude (60° N) environment. We hypothesize that the timing of arrival to the colony and, consequently, the timing of parturition is delayed by poorer spring conditions and that unfavorable weather conditions (i.e., low environmental temperatures, heavy rain, strong winds, etc.) during the early growth period slow pup development, in accordance with observations from other studies (e.g., Hoying & Kunz, 1998; Linton & Macdonald, 2018). Additionally, we explore maternal differences across years and potential adaptations to the challenges of breeding in a high‐latitude environment.

## 2 MATERIALS AND METHODS

### 2.1 Data collection

Data was collected during seven consecutive breeding seasons from 2017 to 2023 in Nittedal, Norway (60.11° N, 10.85° E). A small maternity colony consisting of a total of eight reproductive female *E. nilssonii* with known identities and ages (see Section 2.1.1) roosted in an SW‐orientated/exposed wooden bat box on a garage wall 1.5 m above the ground. The box was equipped with a heating device (135‐watt heater with thermostat) at the top; however, this was turned off on very warm days (air temperatures >~25°C) to avoid overheating. Temperature loggers (iButton, model DS1923‐F5, Dallas Semiconductor Inc.) were placed in the top and bottom of the bat box, recording temperatures every hour across one summer season (from 09th May to 24th July 2020) to monitor the temperature gradient within the box. Upper thermal conditions near the heater ranged from 13°C to 46°C (mean: 29.1°C ± 5.4 SD), while thermal conditions in the bottom of the box were on average 11.9°C colder (± 2.8 SD), ranging from 0°C to 35°C (mean: 17.3°C ± 6.6 SD). The thermal conditions within the box were considered generally suitable for this maternity colony, given that the thermoneutral zone of *E. nilssonii* starts around 28°C (Sørås et al., 2023) and that the bats could choose their preferred roosting conditions from a temperature gradient. See Figure S1 for more information on the temperature conditions. A camera (D‐link DSC‐2670 L) was installed in the box before the breeding season of 2019, allowing real‐time observations through the software mydlink (version 2.11.0, D‐Link Corporation) in addition to motion‐triggered video‐recordings. Infrared sensors (ChiroTec Tricorder 9006) were installed at the entrance of the box before the breeding season of 2020 and recorded the timing of each emergence and return at night.

The bat box was manually checked twice per day throughout the breeding seasons: once in the morning to check for the presence of each individual and to (occasionally; five to 15 times per mother per season) take biometric measurements of each adult (weights to nearest 0.1 g using a digital mini‐weight and forearm‐length to nearest 0.1 mm using dial calipers); and once in the evening after pups were born in order to take daily biometric measures and photographs of their development. When approaching the time of parturition, the box was occasionally checked also during mid‐day to get accurate timing of each birth and to get forearm measurements of newborn pups, although we never separated suckling pups from their mothers. The evening measurements of pups were conducted after the females had left the box to forage at night and were always accomplished as fast as possible (finished within 5 min–10 min per night). Because most of the females in the colony had been hand‐raised as pups (see Section 2.1.1) and therefore were used to eating mealworms, a few mealworms (~1 g per bat) and freshwater were supplied after every morning count as compensation for the disturbance; however, the females that had been born in the box and later returned as part of the colony did not eat mealworms (verified through camera‐observations). All handling was carried out under license from the Norwegian Environment Agency (2023/6818 and preceding project licenses).

Across the study period the eight individual females gave birth to a total of 28 viable pups and one stillborn Siamese twin. We recorded 1–7 breeding seasons per female (average 3.3 ± 2.5 SD). Four females gave birth for the first time as one‐year‐olds, while four females gave birth for the first time as two‐year‐olds.

#### 2.1.1 Individual Identification

Of the eight females, six had been brought in as abandoned pups and were hand‐reared until they reached adult size (under license 2001/450 from the Directorate for Nature Management), upon which they were released in the bat box, but were still supplied with additional mealworms until they left the box in autumn for the upcoming winter hibernation. In consecutive years, these six hand‐reared females returned to the bat box in spring to give birth. The remaining two females in the colony were daughters born in the box that returned the following summers as part of the maternity colony. The identity of each bat was confirmed at the beginning of each breeding season by photographing their wings, using an external flash as a backlight, as patterns of collagen–elastin bundles can be used as an individual identifier (Amelon et al., 2017). We confirmed that this was a successful method for identification in our small maternity colony by investigating cross‐year wing‐photos taken of the two oldest females, which had been ringed as juveniles (under license 06‐3742 from the Norwegian Environment Agency). Given the small number of bats present each year, the identification throughout the summer was afterwards made through measurements of forearm‐length and observations of individual morphological characteristics and was re‐confirmed by wing‐photos taken during the season. Pups were generally born with a few days intervals and were therefore easily identified by their postnatal development stage until they reached adult size, upon which forearm‐measurements, visual morphological characteristics, and wing‐photos were used to confirm their identities. In cases of similar birth dates, non‐toxic nail polish was used on their toenails to differentiate between pups.

#### 2.1.2 Meteorological data

We downloaded weather data with 10 min intervals from the meteorological stations SN4460 (temperature, wind, and rain; 11.7 km from the study site) and SN18700 (barometric pressure; 12.8 km from the study site) through the Norwegian Centre for Climate Services (NCCS). Sunset and sunrise data were obtained through the Time and Date webpage (Timeanddate.com).

### 2.2 Data analyses

We performed all analyses in R (version 4.3.1). The specific model construction and information on dependent and independent variables for each analysis are described in the subsections below. For all analyses, we fitted hierarchical models (mixed models) with individual ID and year included as random effects to account for the lack of independence in our data (individuals and years with repeated measures). The mixed models (linear mixed models (LMMs) or generalized mixed models (GLMMs)) were fitted using the *lmer* or *glmer* functions from the lme4 package (Bates et al., 2015). In all models, only additive effects were tested (i.e., no interactions) except for one model where the gestation stage was tested in interaction with environmental effects (see Section 2.2.2). We based our model selections on Akaike information criterion corrected for small sample sizes (AICc) and model weights using the *dredge* function from the MuMIn package (Barton & Barton, 2015). To avoid collinearity, we performed initial smaller models in cases where several versions of the same variable or highly correlated variables were of interest (e.g., mean, maximum, and minimum temperature values for day‐time or night‐time). We then selected which of the correlated variables to include in the global models based on the AICc values of these simpler models. The fixed effects tested in each model described below are the chosen variables after such initial tests. To disentangle within‐ versus among‐individual effects of explanatory variables in the final models (i.e., after model selection), we applied the mean‐centering methods described in van de Pol and Wright (2009) for distinguishing the effects of within‐individual plasticity versus among‐individual differences.

#### 2.2.1 Arrival phenology

Arrival day and daily presence of each female in the colony were recorded during the morning box checks. To investigate which spring weather conditions that best explained the variation in arrival date to the colony, we fitted LMMs with arrival day in relation to 1st May as the response variable. The fixed effects tested were the onset of spring (the ordinal day when the 10 day smoothed daily temperature is above 0°C for at least 10 days (Fjelldal et al., 2020)) *or* mean temperature conditions for one of three selected time periods: from 1st April to 30th April, from 15th April to 15th May, and 1st May until 31st of May (temperature for each period was tested separately due to correlations). We chose to limit the number of time periods tested due to our small sample size (*N*_obs_ = 28). Total rainfall for each period was also included as a fixed effect in each model. We then performed model selections on each global model, and the AICc of each of the highest‐ranked models were compared to see which spring weather condition that best explained arrival time.

#### 2.2.2 Gestation period

During the gestation period, pregnant females occasionally left the bat box to return a few days later. To test which weather conditions affected these periods of absence, we treated individual presence in the colony during the gestation period as a binary response (present = 1, absent = 0) in GLMMs (family = “binomial,” link = “logit”). Maximum air temperature and total precipitation on the day before the observation were included as fixed effects. We also categorized each presence observation into “early gestation” (≥15 days left until parturition) or “late gestation” (<15 days left until parturition) and tested temperature and rainfall in interaction with gestation stage. We chose to categorize the time left until parturition due to high correlations with the temperature variables and due to the observations of females being absent were made either very early in the gestation period (from 24.5 to 36.7 days left until parturition, mean = 30.9 days ±3.2 SD, *N*_obs_ = 13) or very late (from 0 to 10.7 days left until parturition, mean = 6.7 days ±0.9 SD, N_obs_ = 38).

To assess the effect of arrival time and periods of absence on the gestation duration, we fitted an LMM with the number of days from arrival to parturition as the response variable. Individual arrival days (days since 1st May), the number of days of absence from the colony in the early gestation period, and the number of days missing in the late gestation period were included as fixed effects in the global model.

#### 2.2.3 Parturition and morphologic development

Timing of parturition was closely monitored through the daily box‐checks and with the camera. The morphologic development of the bat pups was monitored through the evening box checks after mothers had left to forage. During these checks, we noted developmental observations (i.e., presence/absence of umbilical cord, opening of the eyes) and took pictures of fur growth.

#### 2.2.4 Size at birth

Variations in body weight and forearm length at birth were tested as response variables in two global LMMs that included age at measurement, sex, age of the mother, whether the mother ate offered mealworms or not, gestation time, birth date in relation to 1st of June, total rainfall and mean temperature the last 3 weeks before parturition as fixed effects. Because mean temperature and total rainfall were highly negatively correlated (−0.82), these variables were tested in separate models. The best models from the two model selections for each response variable were then compared to find the better fit.

#### 2.2.5 Growth curves and most rapid growth period

To describe overall growth curves for *E. nilssonii* pups, we tested three different growth models, these being the logistic, Gompertz, and von Bertalanffy growth models, with either forearm length or body mass as the response variable and the age of the pup as the independent variable. The AICc of each growth model was then compared using the *aictab* function from the AICcmodavg package (Mazerolle & Mazerolle, 2020) to identify the best fit for forearm length and body mass, respectively.

However, the growth in both forearm length and body mass in bat pups contains a rapid, approximately linear growth period before the growth declines. To determine the duration of the most rapid growth period, we performed breakpoint analyses from the segmented package (Muggeo, 2008) on simple linear models with either forearm length and body mass as response variables and age as the independent variable. A breakpoint analysis detects if and where there is a substantial change in the slope of the specified model (Muggeo, 2008), which here would indicate the end of the linear growth period for the pups.

Finally, to investigate the effects on individual growth rates for forearm length and body mass during the most rapid growth period, we constructed global LMMs with average daily growth rates (during the most rapid growth period) for forearm length and body mass as response variables and tested the following fixed effects: sex, age of the mother, whether the mother was eating offered mealworms or not, birth date in relation to 1st of June, total rainfall and minimum air temperatures at night during the most rapid growth period.

#### 2.2.6 Adult body size

Variation in size of pups approaching adult size were explored by fitting LMMs. Two response variables were tested; to assess forearm length, we used the largest recorded forearm‐length for each pup, while for body mass, we used the heaviest individual body weight recorded (before 3 weeks of age, as several pups were not measured after this point in time). Fixed effects included in the global models were sex, age of the mother, whether the mother ate offered mealworms or not, birth date in relation to 1st of June, total rainfall, and mean temperature the three first weeks after birth.

#### 2.2.7 First flight week

The timing of pups becoming volant was observed either on camera or by registering absence during the evening box checks. Emergence time and duration of trips during their first flight week were recorded through manual assessment of video recordings. Pups were differentiated based on size and morphologic development. In cases where pups were born less than a few days apart, non‐toxic nail polish was used on their toenails, to help identify which individual(s) that were left in the box during the evening box‐checks. Usually, only the two first juveniles each year were possible to identify on camera during their first flight week, after which the volant juveniles were not possible to differentiate on camera alone. The differences between adults and juveniles were, however, easily detected on camera at this point in time (based on size and fur characteristics) and allowed us to differentiate between flight patterns (time of emergence and flight durations) of juveniles and adult bats on the same flight nights.

## 3 RESULTS

### 3.1 Arrival phenology

**FIGURE 1:**
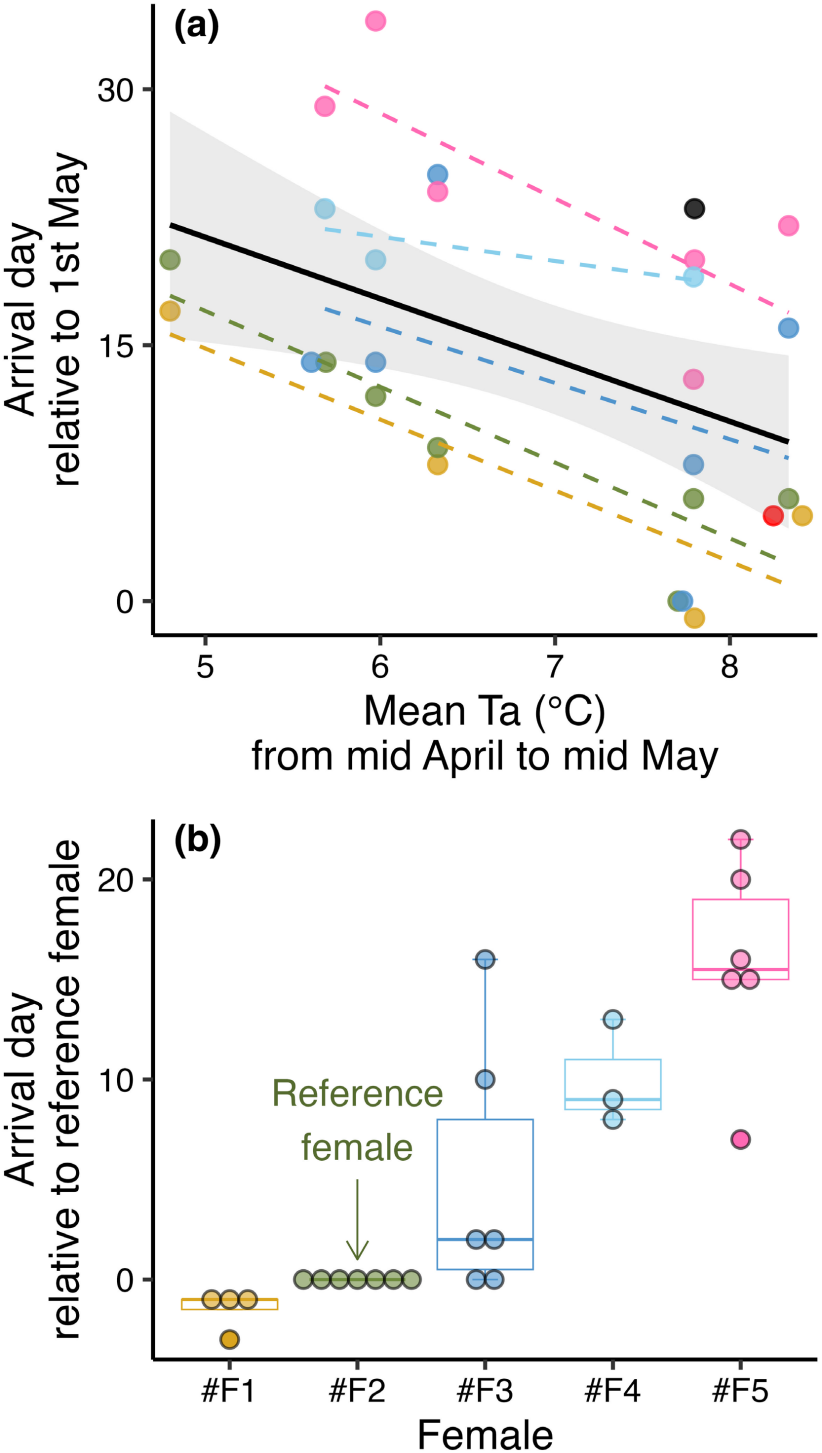
Variation in arrival day at the breeding colony. (a) The effect of increasing mean temperature conditions between 15th April and 15th of May leading to earlier arrival dates in spring, with the black regression line showing the overall effect while different colors indicate different females. (b) Timing of arrival for each female relative to a reference female (*#F2*). Three females were only present for one breeding season and are therefore not shown with individual regression lines or boxplots.

The females generally arrived at the colony in May and gave birth to their pups in June (Table 1). The model that best explained the variation in arrival date in spring included a negative effect of mean temperature between 15th April and 15th of May, which we confirmed was driven by within‐subject effects (Table 2a; N_obs_ = 28). However, although each female showed similar responses of arriving ~4 days earlier per one‐degree increase in mean temperature conditions (Table 2a; Figure 1a), there were strong individual differences in arrival time. Figure 1b illustrates our observations of how each female arrived at certain time intervals when compared to a reference female that was present across all seven breeding seasons, showing that some females arrived generally early while others arrived late relative to each other.

**TABLE 1:** Phenological and developmental observations made across the study period.

Observations	Dates	Time since birth (mean ± sd)	*N*_obs_
Females arrive in the colony	30/04–04/06	−23.6 to −40.4 days (−33.5 ± 5.4)	28
Parturition	07/06–27/06		28
Last obs. of umb. cord^a^	07/06–28/06	1.7–26.3 h (median = 4.6)	24
Pups open eyes	09/06–01/07	1.6–5.4 days (4.1 ± 0.9)	26
Pups' first flight	24/06–13/07	13.1–19.4 days (15.3 ± 1.6)	25
Mothers leave the colony	05/07–03/08	14.1–46.2 days (32.7 ± 8.1)	21
Pups leave the colony	05/07–31/07	14.1–40.4 days (30.6 ± 7.0)	18

^a^ Last observation where the umbilical cord was still attached.

**TABLE 2:** Model results presenting original (undecomposed) and decomposed within‐subject effects from the final best‐fit models (after model selection) explaining: (a) arrival date; (b) presence in colony during gestation period; and (c) gestation period duration (number of days from arrival until parturition).

Variable	Random effects	Fixed effects			
		Original model effects		Within‐subjects effects	
	Variance (SD)	Estimate (SE)	*p* value	Estimate (SE)	*p* value
(a)	Model: Arrival date (days since 1st May)				
*Year*	0.74 (0.86)				
*Female ID*	41.42 (6.44)				
*Residual*	23.06 (4.80)				
Intercept		42.79 (6.67)	<.001	29.70 (32.8)	.39
Mean Ta (April–May)		−3.98 (0.87)	<.01	−4.03 (0.87)	<.01

(b)	Model: Daily presence during gestation (probability)				
*Year*	3.99 (2.0)				
*Female ID*	0.39 (0.62)				
Intercept (early gest)		−2.35 (1.38)	.09	8.69 (4.93)	.08
Late gestation		18.27 (3.40)	<.001	6.09 (7.97)	.45
Max Ta day before		0.38 (0.07)	<.001	0.42 (0.07)	<.001
Max Ta × gestation		−0.80 (0.13)	<.001	−0.85 (0.14)	<.001

(c)	Model: Gestation duration (days from arrival until parturition)				
*Year*	0.84 (0.92)				
*Female ID*	1.32 (1.15)				
*Residual*	1.91 (1.38)				
Intercept		35.79 (1.15)	<.001	31.6 (1.76)	<.001
Arrival day		−0.25 (0.05)	<.001	−0.18 (0.07)	<.05
Days missing (early)		0.76 (0.14)	<.001	0.68 (0.17)	<.01

### 3.2 Gestation period

**FIGURE 2:**
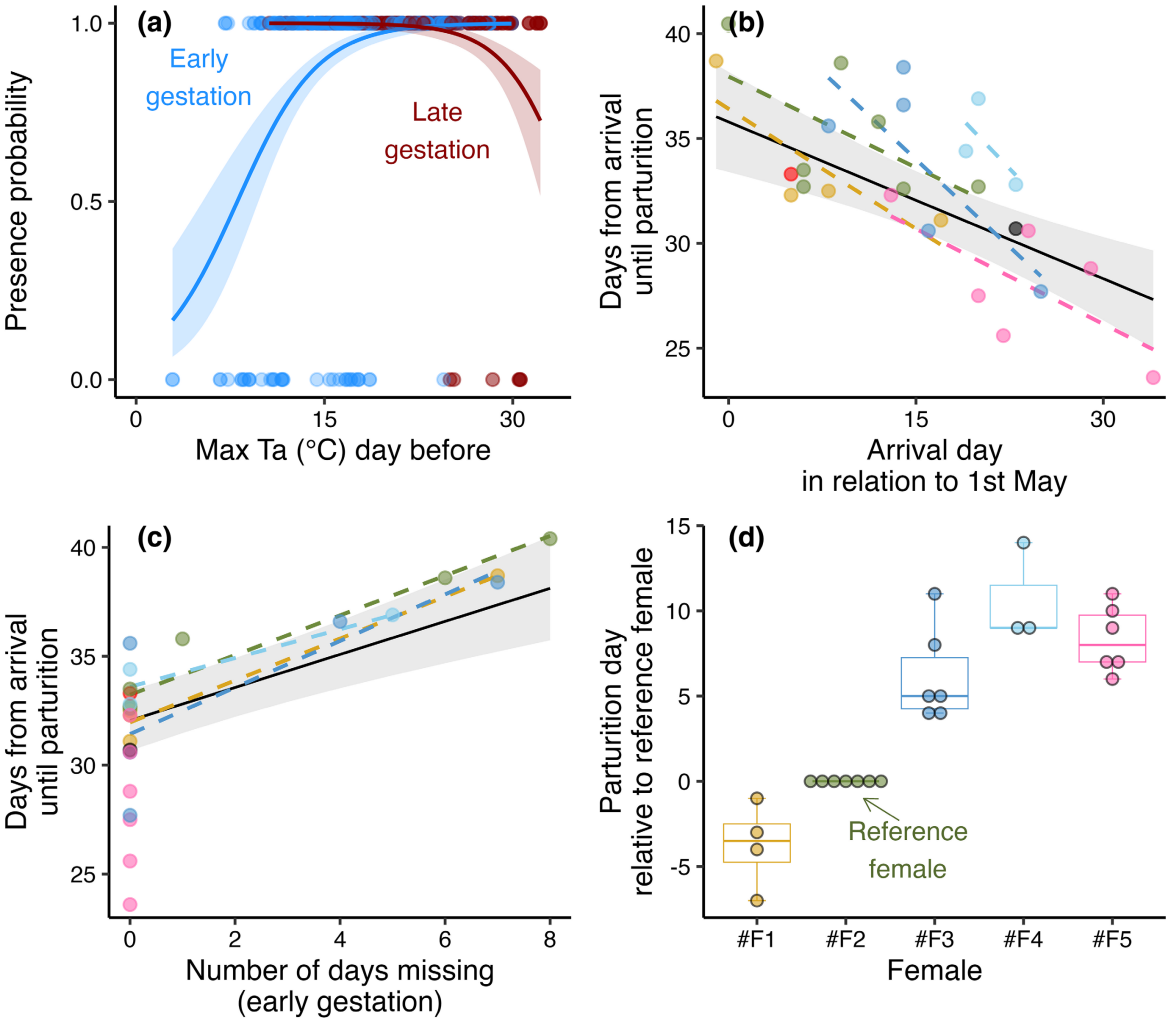
Gestation period dynamics. (a) The probability of a female being present in the colony in relation to maximum temperature the day before, where colder temperatures decreased the presence probability during the early gestation period (blue), while warmer temperatures decreased the probability during the late gestation period (red). (b) The negative effect of arrival date on the duration from arrival until parturition, with the black regression line illustrating the predicted overall effect while different colors indicate different females. (c) The positive effect of absent periods during the early gestation period, with the black regression line showing the predicted overall effect while different colors indicate different females. (d) Timing of parturition for each female when compared to a reference female (*#F2*).

Daily presence probability in the colony during the gestation period was best explained by an interaction effect between maximum temperature the day before and the gestation stage (“early” or “late”) (Table 2b; *N*_obs_ = 874). Colder maximum temperatures led to an increasing probability of females leaving the colony the following night in the early gestation period, while warmer maximum temperatures increased the risk of leaving the colony in the late gestation period (Figure 2a). The effects were confirmed to be driven by within‐subject effects (Table 2b).

The time period from a female first arrived in the colony until giving birth lasted between 23.6 and 40.4 days and was best explained by female arrival date and number of days being absent during early gestation. The predicted gestation duration for females arriving on the mean arrival date (15 days after 1st of May) with 0 days missing was 32.1 days (95% CI [30.7, 33.4]; Table 2c). Later arrivals resulted in reduced gestation duration, which we confirmed was driven by within‐subjects effects (Figure 2b and Table 2c). The periods of absence from the colony prolonged the gestation period with approximately the same number of days as the females were missing, but only during the early gestation period when the absence was caused by cold weather conditions (Table 2c and Figure 2c). Females leaving the box late in the gestation period (in response to warm temperatures) did not prolong the gestation period. We observed similar individual differences in the timing of parturition as we did for timing in arrival (Figure 2d). However, the female that usually arrived last (#F5; Figure 1b) expressed generally shorter gestation periods in the box (pink datapoints in Figure 2b,c) and was therefore not always last to give birth (Figure 2d).

### 3.3 Parturition and morphologic development

**FIGURE 3:**
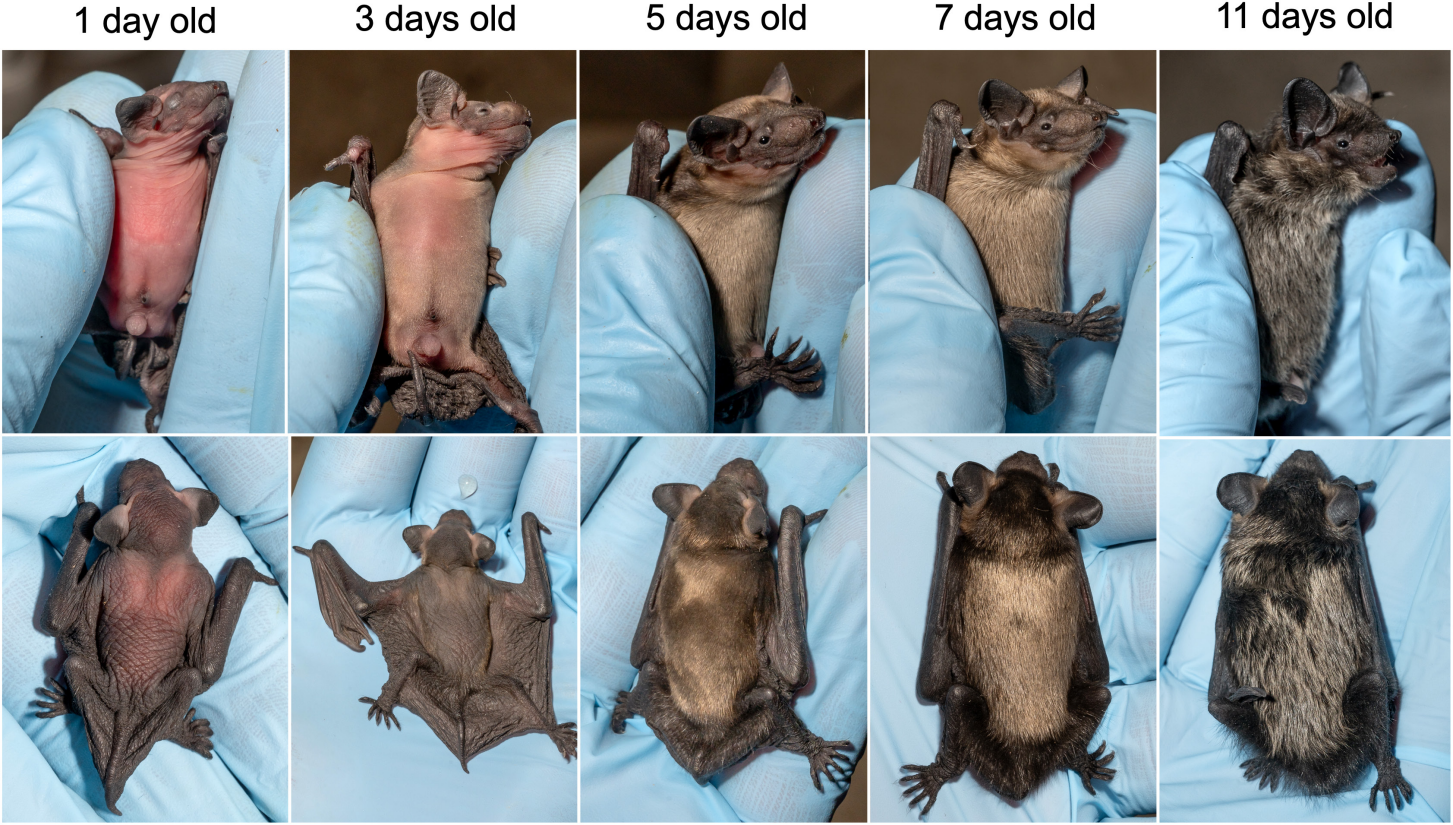
Morphologic development in a male northern bat pup during the first 11 days, demonstrating the gradual fur growth on the abdomen and on the back. The ages are exact (± 2 h). Photos by Jeroen van der Kooij.

Timing of parturition was recorded with high accuracy (from 0 to 6 hours uncertainty, mean: 2.0 h ±2.0 SD, *N*_ind_ = 27), except for on one occasion where a heavily pregnant female left the bat box due to high temperatures and returned to the colony 3 days later with her pup (estimated to have been between ~1.5 and 2 days old based on forearm‐length). A total of 7 female pups, 21 male pups and one stillborn Siamese twin were born during the study period (see Table S1). Newborn pups were on a few occasions, observed with the placenta still connected to the umbilical cord (Figure S2). The last observations of umbilical cords being attached were made from 1.7 to 26.3 hours after birth (median = 4.6 h, *N*_ind_ = 24), although it was only once observed to be attached longer than 24 h. Pups opened their eyes when they were between 1.6 and 5.4 days old (mean = 4.1 days ±0.9 SD, *N*_ind_ = 26). The fur growth on abdomen and back during the most rapid growth period is illustrated in Figure 3.

### 3.4 Size at birth

The estimated body mass at birth, when accounting for age at measurement, was 2.2 g (± 0.17 SE, *N*_ind_ = 21, max age at measurement = 1.5 days), where newborn pups, on average, had body masses corresponding to 25.8% (± 0.06 SD, *N*_ind_ = 12) of their mothers' mean postpartum body masses (each female was measured multiple times throughout the lactation period). Age at the time of measurements was the only variable explaining the variation observed in body mass (estimate: 0.047 g/h ±0.007 *SE*, *p* < 0.001). The estimated forearm‐length at birth in northern bat pups, when accounting for age at measurement, was 15.7 mm (± 0.39 *SE*, *N*_ind_ = 25, max age at measurement = 0.7 days), with newborn pups on average having forearm‐lengths corresponding to 41% (± 0.03 SD, *N*_ind_ = 16) of their mothers' forearms. The model that best explained variation in forearm length at birth included a negative effect of birth date and a negative effect of total rainfall during the three last weeks before parturition (Table 3a; *N*_ind_ = 25). However, rainfall was heavily negatively correlated with mean temperatures (cor: −0.82, *p* < 0.001), and we can therefore understand increasing rainfall values as colder and wetter weather conditions. Later parturition dates and wetter/colder weather conditions the weeks before parturition thus resulted in smaller‐sized pups at birth (Figure S3), which we confirmed were driven by within‐subjects effects (Table 3a).

**TABLE 3:** Model results presenting original (undecomposed) and decomposed within‐subject effects from the final best‐fit models (after model selection) explaining: (a) forearm length at birth; (b) forearm growth rate during the most rapid growth period; (c) body mass growth rate during the most rapid growth period; (d) maximum body mass during the first 3 weeks.

Variable	Random effects	Fixed effects			
		Original model effects		Within‐subjects effects	
	Variance (SD)	Estimate (SE)	*p* value	Estimate (SE)	*p* value
(a)	Model: Forearm length (mm) at birth				
*Year*	0				
*Female ID*	0.15 (0.39)				
*Residual*	0.71 (0.84)				
Intercept		18.86 (0.60)	<.001	17.71 (0.73)	<.001
Birth date		−0.093 (0.03)	<.01	−0.19 (0.04)	<.001
Total rain last 3 weeks		−0.016 (0.006)	<.01	−0.022 (0.005)	<.001

(b)	Model: Growth rate (FA) during most rapid growth				
*Year*	0.0004 (0.002)				
*Female ID*	0				
*Residual*	0.015 (0.12)				
Intercept		1.68 (0.12)	<.001	2.02 (0.35)	<.001
Sex		−0.15 (0.06)	<.05	−0.15 (0.056)	<.05
Birth date		0.011 (0.004)	<.05	0.014 (0.0062)	<.05
Min Ta at night in June		0.071 (0.02)	<.01	0.074 (0.017)	<.01

(c)	Model: Growth rate (BM) during most rapid growth				
*Year*	0				
*Female ID*	0				
*Residual*	0.0045 (0.067)				
Intercept		0.35 (0.06)	<.001	0.37 (0.15)	<.05
Sex		−0.090 (0.03)	<.01	−0.065 (0.03)	<.05
Birth date		0.0056 (0.002)	<.05	0.014 (0.003)	<.001
Min Ta at night in June		0.031 (0.009)	<.01	0.029 (0.007)	<.001

(d)	Model: Maximum BM (<3 weeks)				
*Year*	0.13 (0.36)				
*Female ID*	0.0098 (0.099)				
*Residual*	0.35 (0.59)				
Intercept (eating MW)		9.50 (0.27)	<.001	8.75 (0.41)	<.001
Mothers not eating MW		−1.25 (0.28)	<.01	−1.20 (0.24)	<.001
Total rainfall		−0.029 (0.006)	<.001	−0.035 (0.006)	<.001

*Note*: MW denotes “mealworms”.

### 3.5 Growth curves and most rapid growth period

**FIGURE 4:**
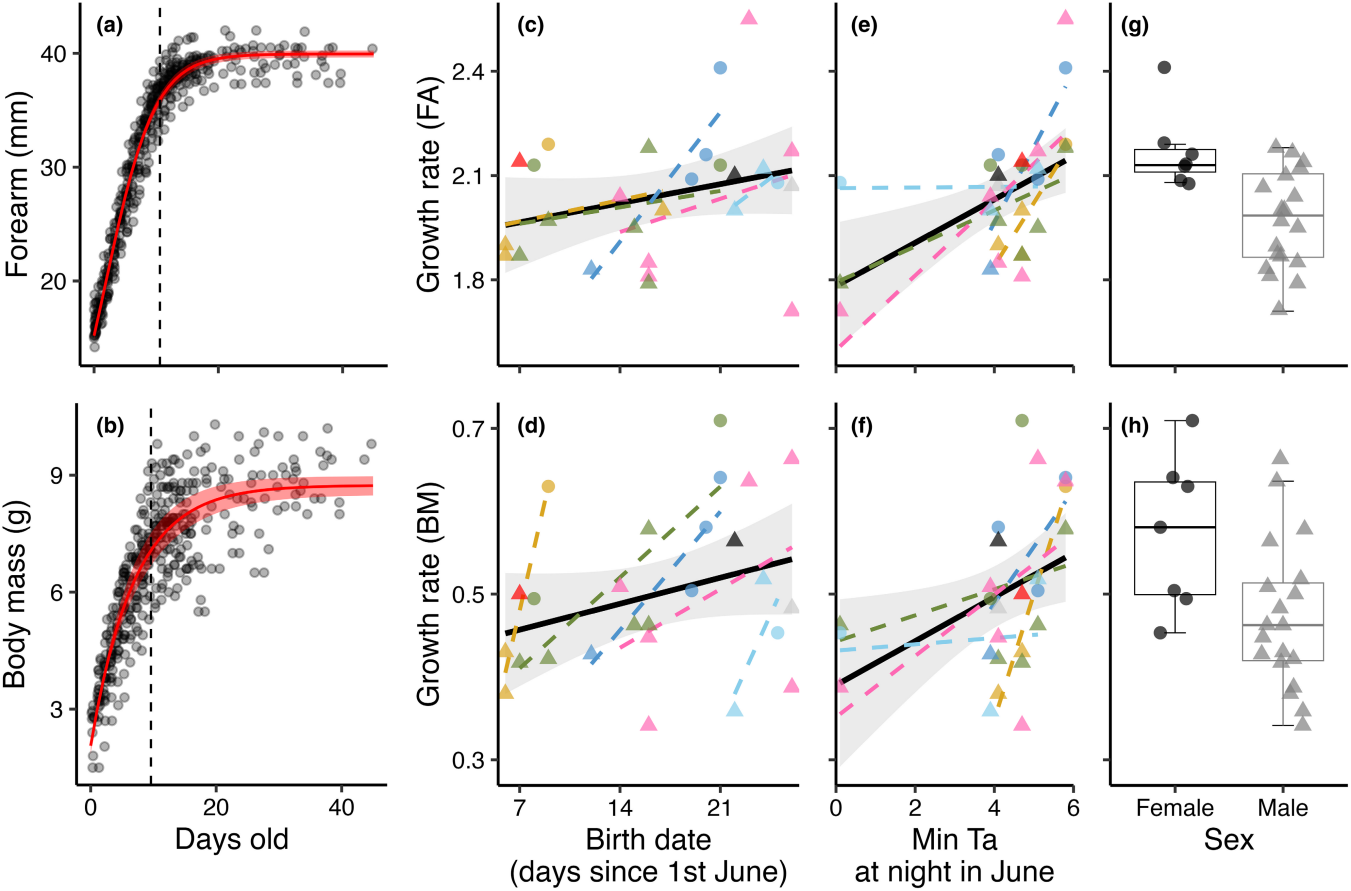
Postnatal growth curves and effects on individual growth rates during the most intense growth period in northern bat pups. (a) Forearm length in relation to age with estimates from the logistic growth model shown as a red line (confidence intervals included as shaded light red). (b) Body mass in relation to age with estimates from the von Bertalanffy growth model shown as a red line. Dashed lines indicate the breakpoint estimates, marking the end of the most rapid growth period. The variation observed in individual growth rates during this period was explained by (c,d) a positive effect of birth date, (e,f) a positive effect of minimum nightly temperature in June, and (g,h) a sex effect, with female pups expressing higher growth rates than male pups. Different colors indicate different mothers, circles mark female pups, and triangles mark male pups.

The logistic model best explained the growth in forearm length (*N*_obs_ = 450, *N*_ind_ = 26) with an equation: FA (mm) = 39.945/1 + e^−0.253 * (days old−1.925)^ (Figure 4a), while the von Bertalanffy model best‐explained body mass increase (*N*_obs_ = 424, *N*_ind_ = 26) with an equation: BM (g) = 8.740 * (1 − e^−0.144 * (days old+1.862)^) (Figure 4b). Model results can be found in Table S2, along with figures of individual growth for forearm length (Figure S4) and body mass (Figure S5) in each pup.

The breakpoint estimate indicating the end of the fastest growth period for forearm length was at 10.6 days (± 0.13 SE) and for growth in body mass at 9.6 days (± 0.3 SE). During this period, starting at birth, the growth is approximately linear, and the model that best explained the variation in individual growth rates during the most rapid growth period (for both forearm and body mass) included sex, date of birth, and minimum nightly temperature conditions in June (*N*_obs_ = 26). For both forearm length and body mass, female pups had higher growth rates than males, and later birth dates and warmer nights in June led to higher growth rates, with all effects being driven mainly by within‐subjects effects (Figure 4c–h; Table 3b,c). The within‐subject effect was considerably stronger than the original non‐decomposed effect for the effect of birth date on body mass growth rate (Table 3c), as seen in the steeper individual regression lines per mother in Figure 4d.

### 3.6 Adult body size

Forearm length for pups approaching adult size varied from 37.4 mm to 42.0 mm (mean: 39.7 mm ± 1.03 SD, *N*_ind_ = 26). None of the tested fixed effects explained the observed variation in adult forearm size, which had an estimated value of 39.7 mm (± 0.2). Maximum individual body mass for pups being less than 3 weeks old ranged from 6.2 g to 10.3 g (mean: 8.3 g ± 1.1 SD, *N*_ind_ = 27). The variation in maximum body mass was best explained by a negative effect of total rainfall during the first 3 weeks after birth, and an effect of whether the mothers ate offered mealworms or not, with mealworm‐eating mothers having heavier pups (Table 3d).

### 3.7 First flight week

**FIGURE 5:**
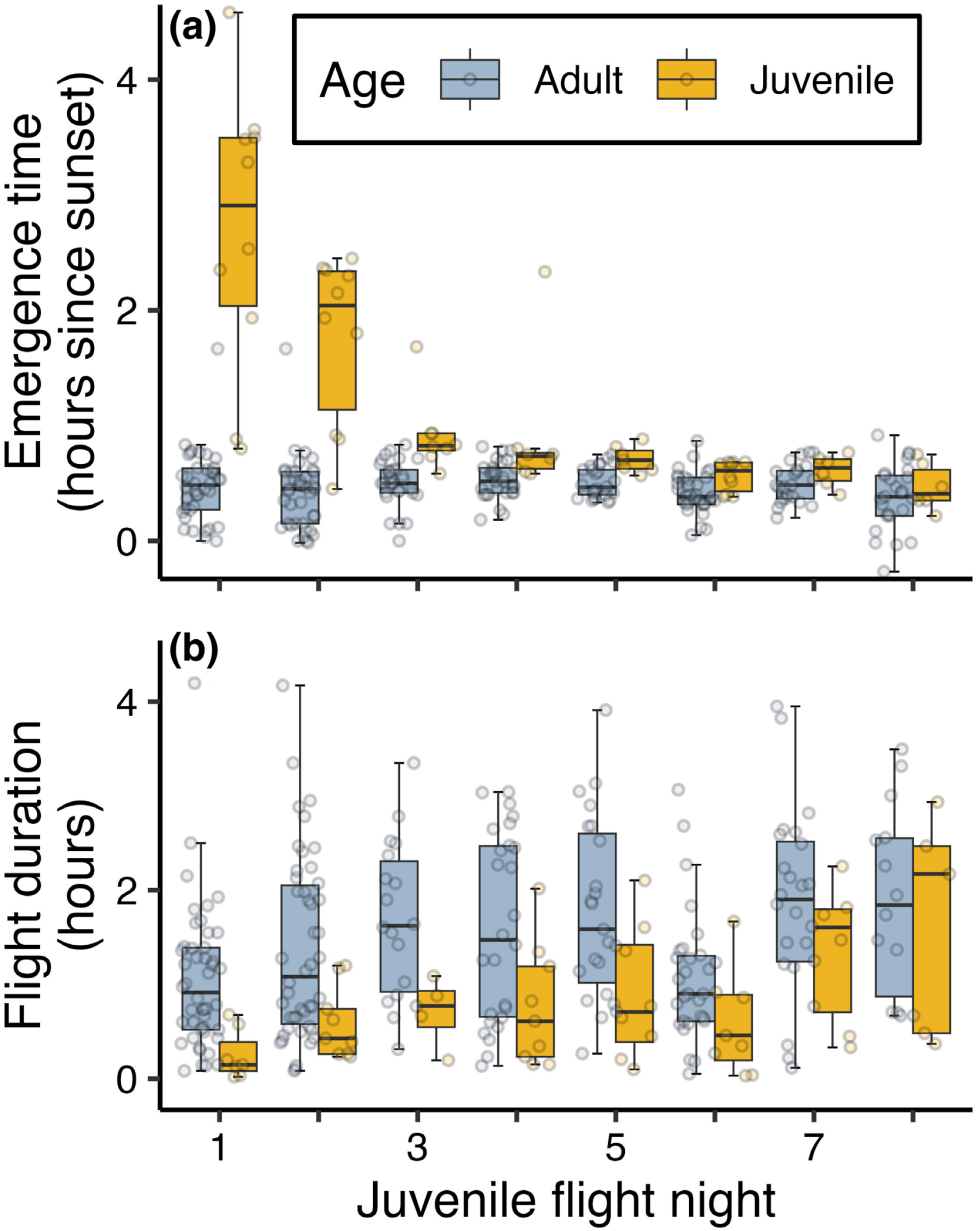
Juvenile flight patterns (yellow) compared to adults (blue) across the juveniles' first flight nights in regard to (a) emergence time (number of hours since sunset) and (b) flight duration (hours).

The age at first flight ranged from pups being 13.1 days to 19.4 days old (mean age: 15.3 days ±1.6 SD, *N*_ind_ = 25). On their first flight night, juveniles emerged between 48 min to 4 h and 35 min after sunset (mean: 2.7 h ±1.2 SD, *N*_ind_ = 10), while adult individuals, for comparison, emerged between 0 min to 1 hour and 40 min after sunset (mean: 28.5 min ±18.6 SD, *N*_obs_ = 38) on the same nights (Figure 5a). Juveniles delayed their emergence time compared to adults also on their second flight night but gradually approached the emergence time observed in adults across the week after their first flight (Figure 5a and Table S3). Furthermore, the duration of the juvenile flights on the first flight night was considerably shorter compared to adults on the same nights, ranging from 1.3 min to 40.6 min in duration (mean: 15.3 min ±15.8 SD, *N*_obs_ = 7) for juveniles and from 5 min to 252 min in duration (mean: 62.7 min ±45.2 SD, *N*_obs_ = 46) for adults. On nights thereafter, the juvenile flights increased gradually in duration until they approached adult flight durations after about a week after their first flights (Figure 5b and Table S4).

### 3.8 Notes on mortality

One male pup died of unknown causes when he was 8 days old, while one female pup never returned from her first flight at the age of 14 days. Because males disperse from the breeding colony as juveniles, the survival rates of male pups after their first and/or subsequent winters cannot be evaluated in our study. Of the six female pups that left the colony after the breeding season, two never returned to the colony, while three returned to the box after surviving their first winter. Two of these returning female pups produced pups of their own in the following years, while the third pup returned as an unreproductive one‐year‐old in the final year of the study (Table S1). The last female pup was born during the final year of the study and can, therefore, not be evaluated.

## 4 DISCUSSION

Here, we present evidence of individual variation in breeding phenology in a high‐latitude study population of *E. nilssonii*, with strong and consequent differences observed between females across years. We further describe apparent adaptations to the short breeding season associated with a northern latitude environment in the postnatal development of *E. nilssonii*: the pups were born big (with ~41% of maternal forearm‐lengths), grew fast (most rapid growth period occurring within 11 days), and became volant already after 2 weeks. However, both reproductive females and their pups were strongly affected by environmental conditions (i.e., rainfall and temperature) throughout the breeding season, with colder and wetter springs or summers resulting in delayed reproduction or reduced postnatal growth.

Although spring temperatures affected the arrival time of all females equally (i.e. similar individual plasticity), some females were consistently early while others were consistently late when compared to each other, which in turn affected the individual timing of parturition. These observations support the indication of among‐individual differences observed in parturition dates for *M. lucifugus* in the study by Sunga et al. (2023). The cause of variation in individual arrival‐ and parturition time remains unknown but could perhaps be influenced entirely or partly by different individual strategies (i.e., variation in personalities (Dingemanse et al., 2010)) existing along the behavioral continuum of initiating gestation early versus late. Females that give birth early face a more stochastic environment, while females giving birth late face reduced time to raise pups before the upcoming mating season. In our study, we observed that females arriving early more often were found to leave the box during the early gestation period in response to cold temperatures, although this prolonged the time until parturition. Females arriving late relative to other individuals avoided most of these cold snaps, but we observed that females arriving later at the colony, both relative to other individuals and relative to their own mean arrival dates, had shorter gestation periods in the box. This aligns with the observations made in a captive population of *Nyctalus noctula* (Zukalova et al., 2022), where females that emerged later from hibernation expressed shorter gestations. However, because we do not know the overwintering sites of each bat, the between‐individual variation in arrival times could be due to different temperature conditions at their overwintering sites and/or different travel distances between the hibernacula of individuals and the joint summer roost. The variation in gestation duration in the box could perhaps be explained by females arriving at the box at a later stage in their pregnancy, although the reason for why females would arrive at different gestation stages at the individual level is unclear.

In temperate environments, poor spring conditions can drastically delay the breeding season and impose long‐term detrimental fitness‐consequences at the population level in bats (e.g. Frick et al., 2010; Linton & Macdonald, 2018; Lučan et al., 2013). We found that at the individual level, there were signs of potential compensation against such effects in *E. nilssonii*; while pups born later in the season were born smaller, they expressed higher growth rates than pups born earlier. This response was substantially stronger (i.e., had a larger effect size) as a “within‐mother” effect for the growth rate of body mass in pups than as an overall response to birth date (Figure 4d, Table 3c). This could suggest that the effect was caused mainly by females compensating for late parturition, for example, by increasing daily food intake (Kurta et al., 1989; Stebbins, 1977) and nursing effort of pups, rather than by an improvement in overall food availability, in which case we would expect the general birth date effect to be equal to or stronger than the within‐subjects effect. Although higher growth rates in bat pups have been reported as a response to shorter breeding seasons across populations (Kunz & Stern, 1995), this has never before been shown to apply at the individual level within bat populations. However, this observation was only true for the growth rate of body mass in our data; the increased growth rate of forearm‐length showed similar effects against birth date both between and within mothers (Figure 4c, Table 3b).

Weather conditions and food availability during spring and summer are already frequently reported as main drivers of intraspecific variation in breeding phenology (Arlettaz et al., 2001; Linton & Macdonald, 2018; Lučan et al., 2013; Matthäus et al., 2023; Ransome & McOwat, 1994; Rydell, 1989a), reproductive success (Burles et al., 2009; Linton & Macdonald, 2018; Lučan et al., 2013; Stapelfeldt et al., 2022) and postnatal development in bats (Hood et al., 2002; Hoying & Kunz, 1998; Koehler & Barclay, 2000; Mundinger et al., 2021). In our study population, environmental conditions during spring and summer influenced both breeding phenology and postnatal development despite the box being heated and a few mealworms being supplied daily. Lower temperatures and rainier conditions delayed arrival time to the colony, reduced daily presence probabilities in the bat box during early gestation, decreased forearm length in pups at birth, decreased daily postnatal growth rates, and resulted in lighter juvenile body mass in three‐week‐old pups. However, we did not detect any environmental, phenological, or maternal effects on the variation in final juvenile forearm‐length, which has been demonstrated in other studies with potential long‐term fitness implications (Mundinger et al., 2021).

Colder temperatures in the early gestation period increased the chances of pregnant females leaving the colony; however, we observed that warmer temperatures in the late gestation period also had a negative effect on daily presence probabilities. In the early stages of pregnancy, we expect that females left the heated bat box for more suitable roosting conditions to enter torpor until weather conditions improved (Willis et al., 2006). This was supported by our findings that absence during early gestation prolonged the gestation duration with approximately the same number of days. However, the observations of pregnant females leaving the box in response to high temperatures, despite the heating being turned off on such days, likely is a result of the roosting conditions reaching inhospitable levels in the colony (Bartonička & Řehák, 2007). Absence in response to high temperatures did not prolong gestation duration, and one female gave birth while absent from the maternity roost before returning with her (estimated) 2‐day‐old pup. Although such roost‐switches can inflict extra costs on heavily pregnant females, highlighting the importance of proper bat box designs (Tillman et al., 2021), a high latitude climate is perhaps not warm enough to result in artificial roosts becoming ecological traps, as was observed for bats in the Mediterranean (Flaquer et al., 2014). Still, in light of ongoing climate change, the design and placement of bat boxes at high latitudes should also be considered given the potential lethal consequences of overheating (Crawford & O'Keefe, 2021).

Across the seven breeding seasons a total of 28 viable pups were born, of which only seven females, resulting in a sex ratio of 3:1 in favor of male pups. Because the female pups were born over years to different mothers (see Table S1) we could not detect any apparent cause for this strong bias towards male pups. No timing differences between sexes were apparent in our data, but we observed that female pups expressed higher growth rates than male pups during the most rapid growth period, although we did not find a sex effect on the final size in juveniles reaching adult sizes. The elevated growth rates could suggest that female pups are perhaps more costly to produce, particularly when faced with the short breeding season at this high latitude, and could be a more careful investment than producing a male pup (Barclay, 2012). However, these speculations will remain untested in our study system.

Our observations of pups flying for the first time as early as 13 days after birth supports the estimated timing of volancy in *E. nilssonii* (Rydell 1989a, Rydell 1989b; earliest volancy recorded 12–17 days after hearing the first baby isolation calls), and is, to our knowledge, the earliest age of volancy recorded in any bat species to date. Volant pups approached adult flight patterns (emergence time and flight durations) by the end of their first flight week, which aligns with observations made of *M. lucifugus* by Buchler (1980).

Although the bats in our studied colony are free‐ranging, they may differ from wild bats in several ways; northern bat females are highly philopatric, like many other temperate zone bat species, which impacts the genetic structure of breeding colonies (Moussy et al., 2013). Because six of the studied females were brought in as pups from different colonies across the country, the genetic variation among the bats is likely larger relative to that found in natural breeding colonies, which could inflate the individual differences we have observed between females in this study. Our observed breeding colony also consists of fewer individuals than what is usually observed in this species (10–80 adults; Rydell, 1993); however, the benefits of social thermoregulation found in larger colonies are likely less pronounced here, because of the heating installed in the bat box. Finally, we acknowledge that the daily disturbance to the colony could potentially impact individual breeding efforts; however, this is a colony consisting of six hand‐reared females, while all colony‐born pups were exposed to handling and box‐checks since birth. We, therefore, expect this colony to be more resilient to the frequent disturbance than wild colonies and thus a suitable study system for this type of day‐to‐day monitoring. This is supported by our observations of breeding females returning interannually to the box and staying for the entire breeding season despite the handling regime.

### 4.1 Conclusions

The day‐to‐day monitoring of individual‐level breeding phenology and postnatal development in *E. nilssonii* presented in this study provides three main novel insights: firstly, we show evidence of strong and consistent maternal differences in breeding phenology in bats across years. Secondly, we show how growth rates are higher in pups born late (relative to mean parturition dates for each female). Thirdly, we show that this population of *E. nilssonii* produces large, fast‐growing pups with the age of volancy corresponding to that found in Swedish *E. nilssonii* colonies, which is the earliest recorded volancy currently observed in any bat species. Our observations offer potential insights to how *E. nilssonii* is adapted to reproducing in high‐latitude environments and, thus potentially how it has become the northernmost breeding bat species in the world.

## AUTHOR CONTRIBUTIONS

**Mari Aas Fjelldal:** Conceptualization (equal); formal analysis (lead); investigation (lead); methodology (lead); writing – original draft (lead). **Jeroen van der Kooij:** Conceptualization (equal); data curation (lead); funding acquisition (lead); investigation (supporting); methodology (supporting); resources (lead); writing – review and editing (lead).

## FUNDING INFORMATION

MAF was funded by the Wihuri Foundation (grant number 00230067), and the Norwegian Environment Agency funded the operation of the national rehabilitation center for bats, managed by JvdK.

## CONFLICT OF INTEREST STATEMENT

We declare no conflict of interest.

## Supporting information


Data S1.

## Data Availability

The data are available online at the following repository: https://doi.org/10.5061/dryad.8sf7m0czk.
